# Tranexamic acid lowers transfusion requirements and hospital length of stay following revision total hip or knee arthroplasty

**DOI:** 10.1186/s13037-021-00295-5

**Published:** 2021-05-11

**Authors:** Arianna L. Gianakos, Bishoy N. Saad, Richard Haring, Luke G. Menken, Sherif Elkattaway, Frank A. Liporace, Richard S. Yoon

**Affiliations:** 1 Harvard-Massachusetts General Hospital, Boston, Massachusetts USA; 2 Divison of Orthopaedic Trauma & Adult Reconstruction, Department of Orthopaedic Surgery, City Medical Center-RWJBarnabas Health, 377 Jersey Ave, Suite 280A, NJ 07302 Jersey City, USA; 3 Vanderbilt University Medical Center, Nashville, Tennessee USA

**Keywords:** Tranexamic acid, TXA, Revision total hip arthroplasty, THA, Revision total knee arthroplasty, blood conservation, TKA

## Abstract

**Backgroud:**

Intravenous tranexamic acid (TXA) has been shown to reduce blood loss in patients undergoing total joint arthroplasty without systemic complications. There is limited evidence of its effectiveness in revision procedures. This study evaluated intravenous TXA effect on blood loss, transfusion rates, and length of hospital stay in revision joint replacement.

**Methods:**

One-hundred revision total joint arthroplasty patients were retrospectively reviewed [44 revision total hip arthroplasty (THA) and 54 revision total knee arthroplasty (TKA)] who underwent surgery from 2013 to 2016. Fifty-four revision joint patients (23 THA and 31 TKA) received intravenous TXA intra-operatively, while 46 revision joint patients (23 THA/TKA) did not. Primary outcome measures were blood loss, transfusion rates, and length of hospital stay.

**Results:**

The mean blood loss difference between revision THA patients who received TXA vs. not receiving TXA was 180ml in revision THA patients (p < .005). Mean length of hospital stay was 6 days in non-TXA vs. 3 days in TXA patients (p < .001). Eighteen patients received transfusions in the non-TXA revision TKA group compared to nine patients in the TXA revision TKA group (p < .001). Average length of hospital stay was 5 days in the non-TXA revision TKA group compared to 3 days in the TXA revision TKA group (p < .003). There was no increased risk of thromboembolic complications in TXA groups for either procedure.

**Conclusions:**

Intravenous TXA reduced length of hospital stay in both revision cohorts, decreased blood loss in revision THA and decreased the rate of transfusion in revision TKA without an increase in thromboembolic complications.

**Level of Evidence:**

Level III (Case-control study)

## Background

The number of total joint arthroplasty procedures performed each year continues to rise, leading to a consequential increase in the need for revision arthroplasty. It has been projected that by 2030, the rates of primary and revision arthroplasty will increase by 174 and 137 %, respectively, compared to the year 2005 in the United States [1]. Revision surgeries have been associated with higher mortality rates, increased hospital length of stay, an overall increased rate of re-admissions, and an increased rate of blood transfusions when compared to primary surgeries [2, 3]. Perioperative and postoperative blood loss has been a concern in primary arthroplasty, and recent studies have demonstrated that blood loss is even greater after a revision procedure [4]. In general, revision surgery is a more complex operation due to extensive bone loss caused by implant removal as well as fibrotic scar tissue formation seen during the exposure as a result of the primary surgery. This often leads to an increase in blood transfusions post-operatively due to difficulties in achieving adequate hemostasis which results in acute blood loss anemia [5]. Transfusions have been associated with complications including immunosuppression, infections, accidental mismatch reactions, transfusion-related acute lung injury, cardiovascular dysfunction, and even death [6]. Blood conserving techniques have been utilized to reduce the need for transfusion such as controlled intraoperative hypotension, regional anesthesia, tourniquet use, and autologous blood transfusions. However, these methods have not been proven to effectively reduce intraoperative blood loss. With an ever-increasing number of revision arthroplasty cases, the financial burden on the medical system will continue to increase as a result [7].

Tranexamic acid (TXA) is an anti-fibrinolytic agent that functions by reversibly binding to the lysine-binding sites of plasminogen, which in turn prevents plasminogen from binding to fibrin. Since plasminogen cannot bind to fibrin, the blood clot is stabilized, promoting an environment to maintain hemostasis [8]. TXA has become a popular blood conservation strategy and has shown to reduce the need for transfusions postoperatively after primary and revision arthroplasty procedures [9–11]. Both intravenous (IV) and topical TXA have shown effective results in decreasing the rates of transfusions and keeping hematocrit levels stable post-operatively [12, 13]. Improved hemostasis has been found with a second dose given at the time of closure without increasing the risk of thromboembolic events [14]. Although the literature has demonstrated a reduction in the number of allogenic blood transfusions, post-operative hemoglobin level drop, and reducing overall blood loss with the use of TXA in revision surgery, the evidence of its effectiveness in reducing length of hospital stay has not been as well established [8, 10, 11, 15–17].

The aim of this study was to (1) determine if intravenous TXA during revision total hip arthroplasty (THA) and revision total knee arthroplasty (TKA) reduces postoperative bleeding, transfusion rates, and length of hospital stay; (2) determine if TXA was associated with an increase in postoperative complications; (3) identify clinical parameters associated with an increased risk of blood transfusion.

## Methods

### Study Design

This was a single-center retrospective cohort study where all surgical procedures were performed by a single attending orthopedic joint reconstruction surgeon. The protocol was approved by the hospital’s Institutional review board.

### Study population

Registry review yielded 46 (mean age, 69 years) and 54 patients (mean age, 66 years) that met inclusion criteria by undergoing revision THA and revision TKA between January 2013 and December 2016, respectively (Table 1). All revision arthroplasty patients were identified using CPT codes for revision total hip or knee arthroplasty (THA-27134, 27137, 27138; TKA-27486, 27487). Exclusion criteria consisted of patients having a primary arthroplasty or revision surgery for fractures/infections, patients on lifelong anticoagulation or patients who had a thromboembolic event within the 12 months preceding surgery.

**Table 1:** Total Cohort Demographics/Post-operative outcomes

Variable		Non-TXA	TXA	P-value
n=		46	54	
Gender	*Male*	16	20	0.84
	*Female*	30	33	
**Mean Age**		71.0 ± 11.5	66.0 ± 10.3	0.03
Mean BMI		30.4 ± 6.6	30.1 ± 5.2	0.81
**Mean ASA**		2.8 ± 0.6	2.5 ± 0.6	0.01
Pre-op Hb		11.4 ± 2.0	12.1 ± 1.7	0.07
**Total Blood Loss**		348.9 ± 219.7	253.6 ± 152.2	0.02
**Length of Stay (days)**		5.7 ± 3.3	3.5 ± 1.8	< 0.001
**Transfusions (units)**		2.5 ± 2.0	1.2 ± 1.7	< 0.001
Drain outputs (total)		347.4 ± 261.8	273.6 ± 308.0	0.24
Complications		8 (17.4 %)	8 (14.8 %)	0.79

Demographic and preoperative data including date of birth, age at time of surgery, gender, BMI, American Society of Anesthesiologists (ASA) score, medical comorbidities, date of surgery, and preoperative hemoglobin (Hb) and hematocrit (Hct) were collected and recorded. Intraoperative data including dose of TXA, tourniquet use, and total intraoperative blood loss were collected. Postoperative data points included postoperative Hb and Hct, incidence of blood transfusion, units given if patient was transfused, 24-hour drain output, method of DVT prophylaxis, complications/adverse events, and discharge date were collected and recorded. Revision type was quantified by the components removed during surgery. In the knee cohort, there were 29 both component revisions and 2 tibial revisions in the TXA group and 16 both component revisions, 3 tibial revisions, 3 polyethylene exchanges, and 1 patellar only revision in the non-TXA group (p = .07). As for the hip cohort, there were 17 both component revisions, 2 stem only revisions, 2 cup only revisions and 1 head and polyethylene exchange in the TXA group and 13 both component revisions, 6 cup only revisions, and 3 head and polyethylene exchanges in the non-TXA group (p = .14).

### Tranexamic acid protocol

Patients who had a history of a thromboembolic or ischemic event, i.e. pulmonary embolism, deep vein thrombosis, ischemic cerebrovascular accident, acute myocardial infarction, or ischemic retinopathy, did not receive TXA. All other patients who did not meet the exclusion criteria received TXA. One gram of intravenous TXA was administered just prior to incision and a second dose just prior to closure.

### Blood transfusion protocol

Hemoglobin and hematocrit levels were drawn and recorded every morning at 5AM. Two units of packed red blood cells (PRBCs) were transfused in patients with Hb levels < 7 g/dl in the general population or with Hb < 8 g/dl if the patient was symptomatic (shortness of breath, dizziness, hypotension, tachycardia), had a history of cardiac disease, or was over the age of 70 years. If transfusion was required, post-transfusion Hb and Hct levels were also drawn. The total number of units transfused was recorded.

### Statistical analysis

Categorical variables were compared using the chi squared test and continuous variables were analyzed using the Kruskal-Wallis test. Modeling was done using robust linear regression to adjust for the influence of outliers; results were adjusted for all other variables included in the model. Alpha was set at 0.05. All analyses were conducted using Stata 13.1 (StataCorp LLC, College Station, TX).

## Results

A total of 46 revision THA patients (23 in the TXA group and 23 in the non-TXA group) were included in the study with similar demographic characteristics (Table 2). Fifty-four revision TKA patients (31 in the TXA group and 23 in the non-TXA group) were included for analysis (Table 3). In the revision TKA cohort, the TXA group tended to be younger (mean 65.6 years vs. 71.9 years, p = .025) and have a lower ASA class (mean 2.3 vs. 2.8, p < .001) than those in the non-TXA group. Revision joint patients receiving TXA had significantly shorter postoperative hospital stays than those not receiving TXA (THA: 3.2 days vs. 6.0, p < .001, Table 4) (TKA: 3.5 days vs. 5.2, p = .003, Table 5) and after adjustment for covariates, maintained statistical significance.

**Table 2:** Demographics THA

Variable	Non-TXA	TXA	p
Gender			
*Male*	9	6	0.34
*Female*	14	17	
Mean age	69.7	66.5	0.37
Mean BMI	31.8	29.4	0.26
Mean ASA class	3.55	3.68	0.86
Pre-op Hb	11.1	11.3	0.65

**Table 3:** Demographics TKA

Variable	Non-TXA	TXA	p
Gender			
*Male*	7	14	0.272
*Female*	16	17	
**Mean age**	71.87 (61–83)	65.58 (58–75)	**0.025**
Mean BMI	32.84	30.93	0.535
**Mean ASA class**	2.83	2.30	**< 0.001**
Pre-op Hb	11.77	12.53	0.061

**Table 4:** Postoperative outcomes by TXA status THA

Variable	Non-TXA	TXA	p
**Total blood loss**	477.3	297.7	**0.005**
**Length of Stay (days)**	6.0	3.2	**< 0.001**
Postop units transfused (mean)	3.5	2.6	0.098

**Table 5:** Postoperative outcomes by TXA status TKA

Variable	Non-TXA	TXA	p
Total blood loss	217.39	200.52	0.247
**Length of Stay (days)**	5.22	3.48	**0.003**
**Postop units transfused (mean)**	2.5	0.9	**< 0.001**

TXA revision hip patients also had less total blood loss (combined intra-operative and postoperative) than those not receiving TXA (297.7mL vs. 477.3mL, p = .005). After adjustment for covariates, this effect, while substantial, was no longer significant (-98.4mL, p = .143, Table 6). There was no significant difference in postoperative complications or thromboembolic events between the two revision THA groups.

**Table 6:** Effect of TXA and other variables on total blood loss, 24-hour drain output, postoperative length of stay, and transfusion rate adjusted for other covariates. THA

**Variable**	**Regression Coefficient (Unadjusted)**	**p**	**Regression Coefficient (Adjusted)**	**p**
**Total blood loss**				
*TXA*	-130.9	0.012	-98.4	0.143
*Age*	5.9	0.007	6.2	0.059
*BMI*	6.8	0.054	6.7	0.162
*ASA Class*	2.2	0.939	1.9	0.963
*Preop Hb*	-29.9	0.062	-11.2	0.606
*Female sex*	-38.5	0.488	-104.1	0.173
**24h drain output**				
*TXA*	-48.4	0.507	-2.2	0.978
*Age*	2.4	0.457	5.1	0.182
*BMI*	6.1	0.364	0.7	0.923
*ASA Class*	59.4	0.091	73.1	0.118
*Preop Hb*	20.0	0.401	31.1	0.222
*Female sex*	-155.4*	0.033	-116.7	0.194
**Postoperative length of stay**				
***TXA***	-2.38	<0.001	-1.68	**0.032**
*Age*	0.07	0.016	0.01	0.750
***BMI***	0.15	0.009	0.16	**0.005**
*ASA Class*	0.04	0.927	0.02	0.971
*Preop Hb*	-0.31	0.173	-0.41	0.105
*Female sex*	-1.02	0.175	-0.63	0.460
**Postoperative units transfused**				
*TXA*	-0.84	0.103	-0.23	0.615
***Age***	0.08	<0.001	0.07	**0.004**
*BMI*	0.02	0.601	0.02	0.595
*ASA Class*	0.64	0.016	0.57	0.054
*Preop Hb*	-0.44	0.006	-0.22	0.155
*Female sex*	-0.28	0.614	-0.38	0.469

A significant reduction in transfusion rates in patients treated with TXA (9 total transfusions vs. 18, p < .001) in the revision TKA cohort was seen. Robust regression modeling revealed a non-significant reduction in total blood loss among TXA patients (-49.2mL, p = .092) when compared with non-TXA patients after adjustment for other covariates (Table 7). There was no significant difference in post-operative complications or thromboembolic events in the revision TKA groups.

**Table 7:** Effect of TXA and other variables on total blood loss, 24-hour drain output, and postoperative length of stay, adjusted for other covariates. TKA

**Variable**	**Regression Coefficient (Unadjusted)**	**p**	**Regression Coefficient (Adjusted)**	**p**
**Total Blood Loss**				
*TXA*	-30.56	.189	-49.2	.092
*Age*	-0.97	.419	-0.85	.502
*BMI*	2.46	.156	2.34	.239
*ASA Class*	-27.22	.247	-39.44	.127
*Preop Hb*	-1.24	.839	1.29	.845
*Female sex*	17.8	.448	24.80	.350
**24h drain output**				
*TXA*	-45.64	.523	-82.50	.326
*Age*	-1.50	.651	0.14	.969
*BMI*	1.93	.721	3.62	.529
*ASA Class*	-34.26	.579	-62.52	.400
*Preop Hb*	24.66	.166	25.56	.188
*Female sex*	-119.40	.088	-98.36	.204
**Postoperative length of stay**				
***TXA***	-1.24	0.001	-1.06	**0.005**
*Age*	0.00	0.840	-.01	0.652
***BMI***	0.07	0.005	.06	**0.020**
*ASA Class*	0.24	0.384	-.04	0.888
*Preop Hb*	-0.08	0.321	-.03	0.686
*Female sex*	0.21	0.564	-.08	0.806

## Discussion

These results demonstrated a reduction in length of hospital stay with the administration of two doses of intravenous TXA in revision arthroplasty procedures. A significant decrease in overall total blood loss was found in revision THA patients, 297.7mL vs. 477.3mL (p < .005). Previous studies have demonstrated similar findings when evaluating TXA in either primary or revision THA [8, 10, 11, 16, 18]. While this effect was substantial, it was no longer significant (-98.4mL, p = .143) when adjusting for covariates, suggesting that at least a portion of the effect could be explained by differences in the underlying patient population, though low study power may also have played a role. Furthermore, a decrease in transfusion rate in revision TKA who received TXA was found. There was no increase in postoperative complications or thromboembolic events in patients who received TXA in either revision arthroplasty groups. Although the effectiveness of TXA in reducing blood loss has been demonstrated when used in primary arthroplasty, there has been limited literature reporting on its role in revision cases and decreasing postoperative length of stay [19].

Revision surgeries require larger surgical exposures with dissection through fibrotic tissue in order to remove prior implants and place revision components. The tissue tends to be friable creating difficulties in achieving hemostasis and ultimately resulting in increased blood loss [18, 20]. When compared with primary procedures, studies have demonstrated that revision THA may result in up to 2000 mL of additional blood loss and increased transfusion rates have been reported with revision TKA procedures [21]. This acute blood loss anemia secondary to revision procedures can have a systemic effect on the patient and result in tachycardia, hypotension, and an increased risk of myocardial infarction [22, 23]. To counter these effects, transfusions are utilized to aid the patient in achieving homeostasis but they are not without risks of allergic reactions or worse, death [6]. This study demonstrates a reduction in transfusion rates in both revision surgery groups with statistical significance reached in revision TKA who received TXA which coincides with the literature. Park et al. reported a significantly decreased transfusion rate and hemoglobin change in patients who received two doses of IV TXA intraoperatively compared to patients who received no TXA [8]. Smit et al. performed a similar study using a single intraoperative dose of 20 mg/kg of TXA prior to tourniquet release in TKA and reported a significant reduction in hemoglobin loss, transfusion rate, and the volume transfused in patients treated with TXA [15]. Aguilera et al. also demonstrated a reduction in blood loss in patients receiving 1 g bolus dose of IV TXA 15–30 min before pneumatic tourniquet followed by a second dose 60–90 min after the first dose [24]. Additionally, Klement et al. recently showed that TXA use can reduce the risk of periprosthetic joint infection likely due to the decreased need of transfusions in these patients [25].

It is important to note that there has been no established protocol with regards to optimal dosing, timing, and administration of TXA [12, 13]. Although the literature has demonstrated positive effects in primary TKA with administration of intravenous, intra-articular, and oral TXA, there have been no studies evaluating the difference in outcomes in varying doses and schedules including, single bolus dose, repeated doses, continuous infusion, or during and/or after surgery [24, 26, 27]. Patients received two doses of 1 g IV TXA administered just prior to incision and just prior to closure. To date, there has been no consensus on the optimal timing and dosage of IV TXA during arthroplasty procedures. Previous studies have utilized either a standardized dose across patients versus a weight-based dose. Standard dosing typically ranges from 0.5 to 2 g IV TXA. IV TXA typically can be administered at several time points throughout the procedure including at the time of incision, after cementing the prosthesis, just prior to closure, after fascia closure, or after wound closure. In addition, there is no consensus on the number of doses effective for IV TXA administration. Although the optimal administration remains controversial, two intraoperative doses has shown to be effective in reducing blood loss, transfusion rates, and length of hospital stay.

In addition, this study has demonstrated a significant decrease in the length of hospital stay by an average of 2 and 3 days in revision TKA and THA patients who received IV TXA, respectively. Reduction in length of stay with TXA has been previously reported in primary THA and has been helpful in reducing overall healthcare costs [28, 29]. This study also demonstrated no difference in post-operative complications between patients treated with TXA compared to those who did not receive TXA. Therefore, reduction in both transfusion rates and length of hospital stay with no additional increase in complication rate can help reduce the financial burden on the healthcare system. A meta-analysis by Huang et al. evaluated TXA in primary TKA and showed that TXA reduced the number of blood transfusions per patient by 0.78 units; the volume of blood transfusion per patient by 205mL; and the total, intraoperative, and postoperative blood loss by 408, 124, and 214 mL, respectively [30]. Transfusion costs have been estimated to be $84.90 and require 0.13 man-hours to perform [31]. Smit et al. calculated a potential yearly cost savings of $22,300.00 with the introduction of TXA [15]. Similarly, Evangelista et al. calculated a larger yearly saving of $42,825 for THA and $56,644 for TKA. Mahadevan et al. found that patients who received blood transfusion after revision THA spent an additional 6 days in the hospital. They reported an additional hospital cost of £2,400 ($3,300) on prolonged hospital stay alone [32]. Although the evaluation of the cost-effectiveness was not within the scope of this study, the utilization of TXA in revision surgery reduces transfusion rates and decreases length of hospital, which can ultimately reduce overall hospital costs.

Identification of patients who may be at risk for blood loss and the need for transfusion can help determine who should be considered for preoperative blood conservation interventions. This study demonstrates higher transfusion rates with increasing age and increased ASA (American Society of Anesthesia) scores in revision THA patients. Even though these results were not significant when adjusting for covariates, previous studies have identified age and ASA scores as predictors for transfusion and blood loss in patients undergoing THA [33, 34]. Typically, these patients often required transfusions which prolonged overall length of stay. Therefore, aggressive correction of preoperative anemia and proper preoperative medical optimization may help reduce transfusion requirements, hospital stay, and costs. 

There are inherent limitations associated with the design of a retrospective study, however the post-operative outcomes were objective measurements of blood loss, transfusion rates, and length of hospital stay, thereby limiting bias. This study also evaluated the use of two doses of intravenous TXA administered at standard time points during the procedure, therefore it is not possible to report whether dose or timing of administration have an effect on outcomes. Future research should be directed to determine the optimal route, timing, and dosage of TXA in arthroplasty procedures. This study also demonstrated that patients who received TXA tended to have lower ASA grades in revision TKA cases. It is important to underscore that TXA is used in patient groups that exclude patients with a history of DVT or PE, with thromboembolic disease, or heart disease. Therefore, patients with higher ASA scores, demonstrating more moderate to severe systemic disease, may not have been candidates to receive TXA. Additionally, there are confounding factors in the patient population that cannot be ignored. The non-TXA group had a higher average age and a higher average ASA score than their counterparts receiving TXA. Both of these factors have been shown to increase hospital length of stay in patients undergoing joint replacement surgery, previously [35, 36]. It is hard to separate these factors and they should all be considered when optimizing patients for their revision procedure.

## Conclusion

This study demonstrates that administration of two 1 g doses of IV TXA reduces postoperative bleeding in revision THA procedures, rate of transfusion in revision TKA, and length of hospital stay in revision total joint arthroplasty with no increase in thromboembolic complications. TXA should become standard protocol for those indicated patients undergoing revision total joint arthroplasty.

## Data Availability

The datasets used and/or analysed during the current study are available from the corresponding author on reasonable request.

## Abbreviations

ASA: American Society of Anesthesiologist score
THA: Total Hip Arthroplasty
Hb: Hemoglobin
BMI: Body Mass Index
TKA: Total Knee Arthroplasty
Hct: Hematocrit
TXA: Tranexamic Acid
