# Correction to: Tranexamic acid lowers transfusion requirements and hospital length of stay following revision total hip or knee arthroplasty

**DOI:** 10.1186/s13037-021-00313-6

**Published:** 2022-01-07

**Authors:** Arianna L. Gianakos, Bishoy N. Saad, Richard Haring, Luke G. Menken, Sherif Elkattaway, Frank A. Liporace, Richard S. Yoon

**Affiliations:** Department of Orthopaedic Surgery, Divison of Orthopaedic Trauma & Adult Reconstruction, City Medical Center-RWJ Barnabas Health, Jersey, New Jersey 377 USA

**Correction to: Patient Saf Surg 15, 21 (2021)**

**https://doi.org/10.1186/s13037-021-00295-5**

Following publication of the original article [1], the authors noticed that Arianna L. Gianakos (Harvard-Massachusetts General Hospital, Boston, Massachusetts) and Richard Haring (Vanderbilt University Medical Center, Nashville, Tennessee) were not listed as authors when initially published.

A.L. Gianakos is the first author of this article due to substantial contributions to the conceptualization, data collection, analysis/interpretation of the results, writing and editing of the published manuscript

R. Haring is the third author of this article due to contributions to the conceptualization, methodology, statistical analysis and writing of the published manuscript.

The final authorship on this manuscript is: A.L. Gianakos, B.N. Saad, R. Haring, L.G. Menken, S. Elkattaway, F.A. Liporace, R.S. Yoon.

At the time the study was completed, all authors had an affiliation with City Medical Center-RWJ Barnabas Health, Jersey City, New Jersey, USA.

The authors regret this error. In this regard, the original article has been updated.

## References

[1] Saad BN, Menken LG, Elkattaway S (2021). Tranexamic acid lowers transfusion requirements and hospital length of stay following revision total hip or knee arthroplasty. Patient Saf Surg.

